# Rice Performance and Water Use Efficiency under Plastic Mulching with Drip Irrigation

**DOI:** 10.1371/journal.pone.0083103

**Published:** 2013-12-10

**Authors:** Haibing He, Fuyu Ma, Ru Yang, Lin Chen, Biao Jia, Jing Cui, Hua Fan, Xin Wang, Li Li

**Affiliations:** 1 Agricultural College, Shihezi University/Key Laboratory of Oasis Ecology Agricultural of Xinjiang Bingtuan, Shihezi, Xinjiang, China; 2 Agricultural Drought Research Institute of Tianye Group Company, Xinjiang, China; University of Vigo, Spain

## Abstract

Plastic mulching with drip irrigation is a new water-saving rice cultivation technology, but little is known on its productivity and water-saving capacity. This study aimed to assess the production potential, performance, and water use efficiency (WUE) of rice under plastic mulching with drip irrigation. Field experiments were conducted over 2 years with two rice cultivars under different cultivation systems: conventional flooding (CF), non-flooded irrigation incorporating plastic mulching with furrow irrigation (FIM), non-mulching with furrow irrigation (FIN), and plastic mulching with drip irrigation (DI). Compared with the CF treatment, grain yields were reduced by 31.76–52.19% under the DI treatment, by 57.16–61.02% under the FIM treatment, by 74.40–75.73% under the FIN treatment, which were mainly from source limitation, especially a low dry matter accumulation during post-anthesis, in non-flooded irrigation. WUE was the highest in the DI treatment, being 1.52–2.12 times higher than with the CF treatment, 1.35–1.89 times higher than with the FIM treatment, and 2.37–3.78 times higher than with the FIN treatment. The yield contribution from tillers (YCFTs) was 50.65–62.47% for the CF treatment and 12.07–20.62% for the non-flooded irrigation treatments. These low YCFTs values were attributed to the poor performance in tiller panicles rather than the total tiller number. Under non-flooded irrigation, root length was significantly reduced with more roots distributed in deep soil layers compared with the CF treatment; the DI treatment had more roots in the topsoil layer than the FIM and FIN treatments. The experiment demonstrates that the DI treatment has greater water saving capacity and lower yield and economic benefit gaps than the FIM and FIN treatments compared with the CF treatment, and would therefore be a better water-saving technology in areas of water scarcity.

## Introduction

Rice is a main staple food for many people in the world. In Asia, around 700 million people live in rice-growing areas and rice is their main source of calories [1]. Rice crops require substantial amount of freshwater because rice is mostly grown under flooded conditions [2,3]. In Asia, flood irrigation of rice crops consumes more than 45% of total freshwater resources [4]. With rapid industrial and urban development in the area, more freshwater will be required to meet non-agricultural consumption needs. Thus, both total agricultural water consumption and the proportion of freshwater used for agriculture have been decreasing [5]. This will inevitably affect agricultural production of irrigated regions in future, especially irrigated for rice production. Therefore, rice cultivation systems that incorporate water saving methods need to be established to cope with potential water deficit and to ensure that demand for rice continues to be met.

Existing water-saving technologies for rice cultivation can be divided into three groups according to their water-saving capacity. The first group includes the continuously saturated soil cultivation system [6], the rice intensification system [7], and the alternate wetting and drying system [8]. These cultivation systems retain high soil water contents, or in some growth stages, flooded soils, so water losses are high [9]. The second group is known as “aerobic rice”, in which rice, like upland crops, is grown under non-flooded conditions with adequate inputs and supplementary irrigation when rainfall is insufficient [10,11]. Because of a great reduction in seepage, percolation and evaporation, this technology allows for greater WUE and high water saving compared with traditional flooded irrigation [11]. The third group is ground cover rice production systems (GCRPSs) [12,13], which are basically “aerobic rice” systems; they utilize plastic mulching or straw mulching in the cultivation system. Under these ground cover conditions, evaporation can be effectively reduced compared with bare land conditions, so GCRPSs have a greater WUE than “aerobic rice” [13–15]. Tao suggested that plastic mulching cultivation has great potential to substantially save water resources at a high grain yield level compared with traditional flooded irrigation because of the warming and water retention effects of plastic mulching [12]. Therefore, when the stressful factors are water deficits and/ or low soil temperature during the vegetative growth stage, plastic mulching cultivation could be a promising technology to promote rice grain yield formation and WUE [14,16]. 

Studies of water usage of many field crops, such as grapes, cotton, and tomatoes, suggest that different modes of irrigation significantly affect crops growth and WUE, with higher water productivity and higher crop yield obtained under plastic mulching with drip irrigation than under furrow irrigation and sprinkler irrigation [17–19]. However, studies of water-saving technologies in rice production systems have mainly focused on innovations in cultivation systems that incorporate furrow irrigation or sprinkler irrigation [9,16,20,21], with almost no records of rice water-saving cultivation under plastic mulching with drip irrigation. It is important to understand the productivity of rice crops and WUE under the drip irrigation system. In addition, there is little information regarding whether rice, like field crops such as grapes, cotton, and tomatoes and so on, has higher grain yield and WUE under drip irrigation than under furrow irrigation, or whether rice cultivation with drip irrigation has higher grain production potential than that of existing rice water-saving technologies.

Under non-flooded irrigation, the root-zone environment changes from being anaerobic to aerobic. Compared with traditional flooding, fewer roots are distributed in the topsoil layer, while more roots tend to be distributed in deeper soil layers [21–23]. It is also widely believed that the root distribution zone moves upward under drip irrigation when compared with furrow irrigation [18,24]. Moreover, plastic mulching is favorable for rice root growth and development [14,16,25]. However, studies of possible interactive effects of non-flooded irrigation, drip irrigation, and plastic mulching on root growth and distribution are limited. In addition, little is known about the effect of different modes of irrigation on the spatial distribution of roots.

The number of productive tillers per plant plays an important role in the formation of grain yield in rice. Not all tillers are productive and the productivity of tillers mainly depends on cultivars, tillering time, water regime, and plant density [26,27]. Jiang indicated that 27–73% of tillers are productive tillers in traditional irrigation systems and 23–65% of the tillers are productive in aerobic rice cultivation systems [28]. However, in our field investigation from 2010, it was observed that there were few productive tillers under plastic mulching with a drip irrigation cultivation system, and that yield formation mainly depended on the number of main stems per unit area. Unfortunately, clear quantitative data on the percentage of productive tillers and yields from tillers or the main stem under plastic mulching with drip irrigation are not available.

Many studies have investigated existing rice water-saving technologies. The objectives of the present study were to (1) characterize rice productivity under plastic mulching with drip irrigation; (2) compare yield formation and WUE under different cultivation conditions including conventional flooding (CF), plastic mulching with furrow irrigation under non-flooded irrigation (FIM), non-mulching with furrow irrigation under non-flooded irrigation (FIN) and plastic mulching with drip irrigation under non-flooded irrigation (DI).

## Materials and Methods

### Ethics statement

1 The Tianye Group Company was the cooperation research institutions of the project and gave us permission to conduct the study on this site. Therefore, no specific permissions were required for these locations.
2 The field studies did not involve endangered or protected species.
3 The planting density of flooding irrigation referred to field production in Xinjiang province. And the result was from our field investigation in 2010 and 2011 years. No specific permissions were required for this plant density.

### Experimental design and field management

Field experiments were conducted from April to October in 2011 and 2012 at the Agricultural Drought Research Institute of the Tianye Group Company, Xinjiang province, China (44°26.5′N, 86°01′ E). The experimental ﬁeld of non-flooded irrigation in 2012 was adjacent to the ﬁeld used in 2011. The site of the conventional flooding treatments was about 150 m away from the non-flooded irrigation plots in both years. The physicochemical properties of the soil used for conventional flooding treatments were consistent with those of non-flooded irrigation treatments in both years (the conventional flooding plots weren't previously traditional paddy). The soil was heavy loamy (U.S. taxonomy), with 21% clay, 36% silt, and 43 % sand on average over both years, with organic matter, Alkeline-N, Olsen-P, available potassium, pH and soil saturation volume moisture content in the 0–60 cm soil layer being 26.35 mg kg^–1^, 60.83 mg kg^–1^, 25.46 mg kg^–1^, 342.54 mg kg^–1^, 7.30, and 33.71%, respectively.

**Figure 1:**
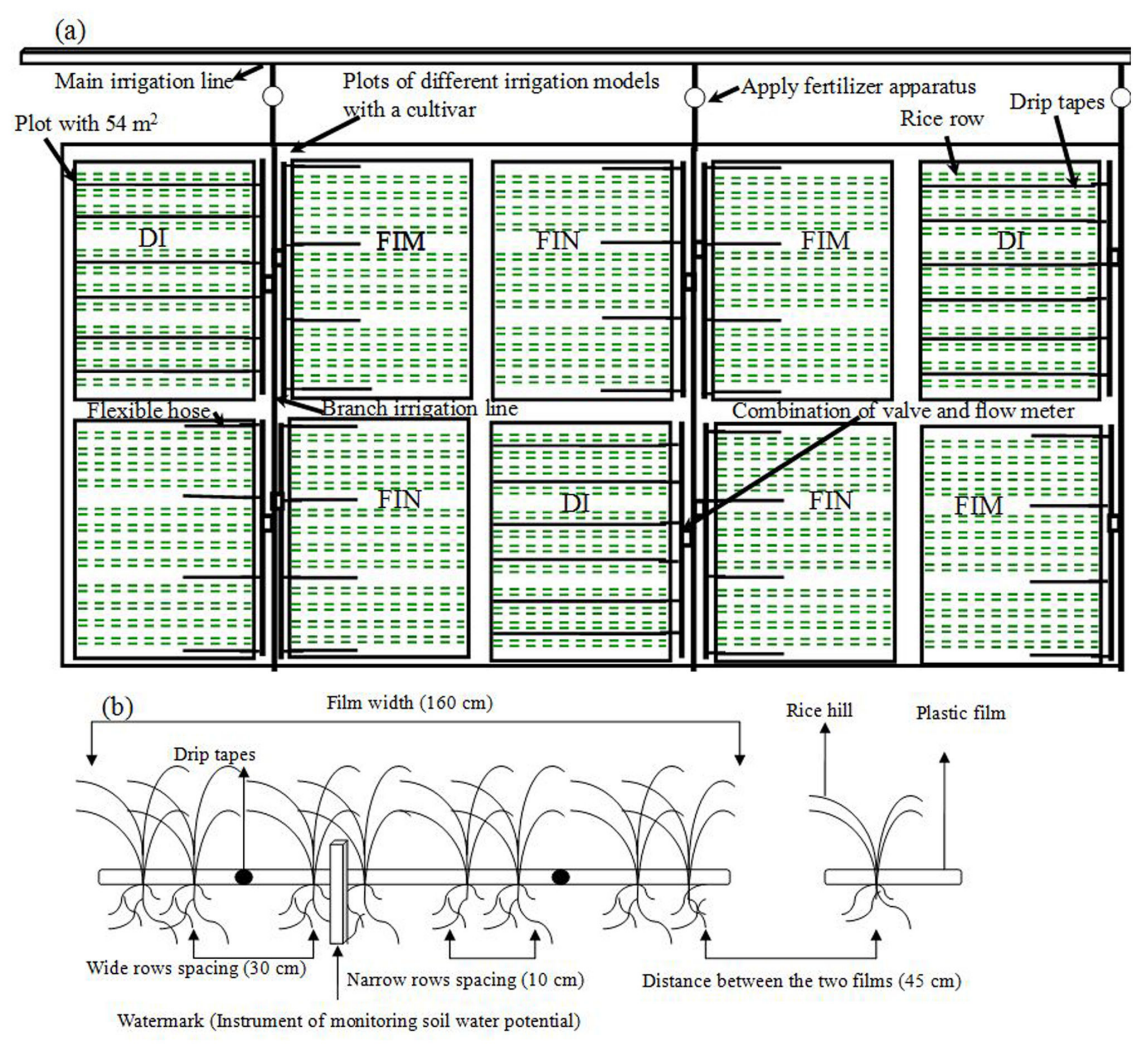
Experimental layouts and sketch map of the planting mode under the non-flooded irrigation treatments (the DI, FIM, and FIN treatments). Experimental layouts (a) and sketch map of the planting mode (b) under the non-flooded irrigation treatments in Shihezi in 2011 and 2012. The DI, FIM, and FIN are plastic film mulching with drip irrigation, plastic film mulching with furrow irrigation, and no mulching with furrow irrigation, respectively.

In 2011, field experiments were conducted using cultivar Ninggeng28 (japonica), which had a good performance under drip irrigation according to our preliminary testing results in 2009 and 2010. Three non-flooded irrigation cultivation treatments were investigated, including the DI, FIM, and FIN treatments, with the experimental treatments arranged in a randomized complete block design with three replicates. In 2012, we added a japonica variety (*Oryza sativa*. L. cv. Xindao17), which is a high-yielding cultivar when cultivated with flooding irrigation under the local ecological conditions. The experiment was laid out in a split-plot design with three replicates, with the cultivation systems set as sub-factors and the varieties as the main factor. All plots in 2011 and 2012 had an area of 54 m^2^ (10 m × 5.40 m). To prevent water exchange between the plots, waterproof membranes were buried to a depth of 60 cm below the soil surface among the plots used for non-flooded irrigation. Dams of 20 cm height were built and covered by plastic ﬁlm for the FIM and FIN treatments to minimize water surface runoff. Each plot in the non-flooded irrigation treatments was covered with a 160 cm wide plastic film before sowing, and eight rows of rice were planted. Then holes were opened on the membrane surface when the rice was sown. Ten seeds were sown on each hill by artificial hand dibbled at a depth of 3 cm on 28 April 2011 and 23 April 2012. Strong seedlings were thinned to six plants per hill after seedling establishment. The planting density was 45.71 hills m^–2^ across cultivars and years under non-flooded irrigation, which the hill spacing in rows was 10 cm and the row spacing configurations were 10-30-10-30-10-30-10-45 cm (10 cm represents a narrow rows spacing, 30 cm a broad rows spacing, and 45 cm the distance between adjacent films. See Figure 1a and b). The plant density and configuration mode of the non-flooded irrigation treatments were mainly based on the preliminary result of high-yielding fields of direct seeding rice under drip irrigation in Shihezi in 2008–2010. Two drip tapes with an emitter discharge rate of 3.20 L h^–1^ and emitter spacing of 0.30 m were laid under the plastic film for the DI treatment. Flexible hoses with a diameter of 2 cm were located on both sides of the plastic film to supply water for the FIM and FIN treatments (Figure 1a). The CF treatment was set as controlled trial across cultivars and years. Under the conventional flooding cultivation system, seeds were sown on the nursery trays on the same day when the non-flooded irrigation plots were sown; twenty-one-day-old seedlings from the nursery trays were transplanted at a planting density of 40 hills m^–2^ (10 cm × 25 cm) with five plants per hill (the planting density referred to field production in Xinjiang province). The CF treatment was arranged in small pools established in 2009 using a randomized complete block design with three replications across cultivars and years, and the area of the each pool was also same with the plot size of the non-flooding irrigation treatments. Because direct-seeded rice has a poor tillering capacity, a bit more seed rate or plant density than the CF system are necessary in field production [27]. Therefore, in this study, we ignored the plant density effect between the direct-seeding technology and the CF system and did a comparative study among different cultivation systems.

At the sowing day, the non-flooded irrigation treatments were irrigated at a rate of 450 m^3^ ha^–1^ to ensure normal germination because a dry direct-seeded system was adopted in our study. No further irrigation occurred until the 3-leaf stage across cultivars and years. Then plastic films in the FIN treatment were removed at the 3-leaf stage to form bare land. The soil water potential was monitored with tensiometer (Irrometer Company, Riverside, CA, USA) buried at 0–20 cm depth below the soil surface between narrow rows (Figure 1b). When the soil water potential in the 0–20 cm soil layer reached –30 KPa (the soil water potential threshold was established by field investigation in 2010), and from the 3-leaf stage to 2 weeks before harvest in the non-flooded irrigation treatments, supplementary irrigation with 30–45 mm and 45–60 mm water was applied in 2011 and in 2012, respectively. Around ﬂowering, the threshold for non-flooded irrigation was set at –10 KPa to avoid spikelet sterility [29]. The CF plots were kept continuously flooding from transplanting to 15 days before harvest, with the water depth maintained at 5–10 cm during the rice growing period. Irrigation water was applied through drip tapes or ﬂexible hoses connected to a brand irrigation line system drawing water from a deep groundwater well for all the water treatments (Figure 1a). The amount of irrigation water was monitored with a ﬂow meter installed in the irrigation pipelines for each plot (Figure 1a) and the total amount of rainfall was calculated from rainfall gauges installed at the experimental site.

 Fertilizers applied were 270 kg N per hectare as urea, 100 kg K_2_O per hectare as potassium chloride, 90 kg P_2_O_5_ per hectare as calcium superphosphate and 30 kg zinc sulfate per hectare. Of these amounts, 10% of the N, and all of the K_2_O, P_2_O_5_, and Zn were applied as a basal fertilizer dressing, with the rest of the N applied in four splits: 20% at the three leaf blade stage, 35% at tillering, 35% at panicle initiation, and the remaining 10% at ﬂowering. 

### Measurements

To measure the aboveground biomass and leaf area index (LAI), plant samples were taken every 14 days from 23 May to 20 September in 2011, and from 20 May to 16 September in 2012 for all treatments. Twelve hills were harvested from each treatment for the CF, FIM, and FIN plots. However, because of non-uniform distribution of water in a horizontal direction in the DI plots, plant performance differences could exist between the near row and far row (Figure 1b), so twelve hills were taken from each row of the DI treatment (If one hill was taken in near row, and another hill beside the sample hill of near row was also taken in far row, and then averaged the two hills, finally, the repeats in the DI treatment were equal to other treatments). The same sampling method was adopted for other parameters in the DI treatment. Samples were separated into leaf blade, stem, and panicle when present. The green leaf area was measured with a LI–3100 leaf area meter (LI COR Inc., Lincoln, NE, USA), and the samples were then dried in an oven at 75°C for at least 72 h and the weights of leaf blade, stem, and panicle were measured. At maturity (15 September in 2011 and 14 September in 2012), 8 m^2^ areas from each plot were harvested to calculate the grain yield and harvest index (HI, the ratio of the ﬁlled spikelet weight to the total aboveground biomass). The grain weight is expressed at 14% moisture content. Finally, matter translocation (MT), matter translocation efficiency (MTE), and contribution of pre-anthesis assimilates to grain weight (CPATG) were calculated: MT = Dry weight of stems and sheaths at anthesis – Dry weight of stems and sheaths at maturity. MTE (%) =MT / Dry weight of stems and sheaths at anthesis × 100. CPATG (%) = Matter translocation / grain weight at maturity × 100. 

At the three-leaf stage (37 days after sowing in 2011 and 39 days after sowing in 2012), thirty hills carrying strong seedlings (sixty hills for the DI treatment) were tagged to investigate dynamic tiller characteristics every seven days for all treatments in both years. Meanwhile, the main stem was labeled by marking it with red paint from the three-leaf stage to heading to distinguish the main stems from tillers. At maturity, nine hills rice (eighteen for the DI treatment) were randomly selected from labeled hills and divided into main stem panicles and tiller panicles for all treatments to determine the yield components of the main stem panicles and tiller panicles. An effective panicle was defined as one having more than five filled grains per panicle. The percentage of ﬁlled grains was deﬁned as a percentage of the number of grains that sink to the bottom of a beaker filled with specific gravity of 1.06 to total spikelets. Spikelets per panicle were the sum of filled and unfilled spikelets. The dry weight (14% moisture content) of one thousand filled spikelets was expressed as the 1000-grain weight. 

At flowering (1 August in 2011 and 3 August in 2012), the roots of three hills (six hills for the DI treatment) in 0–60 cm soil depth, which was averagely divided into three layer, were collected by core sampling for all treatments in both years (the length, width, and height of the sampling soil corer were 20 cm, 10 cm, and 20 cm, respectively. The soil volume theoretically covered the whole hill under the current planting mode for the flooding irrigation and for the non-flooded irrigation). The roots washed with tap water on a 0.50 mm mesh screen were scanned on a ﬂat-bed image scanner (Epson V500, Epson America, Inc., San Jose, CA, USA) and the image was saved in TIF format according to the detailed method described by Kato [21–23]. Then root lengths were analyzed by WinRHIZO commercial software (Regent Instruments, Montreal, QC, Canada). 

The net photosynthetic rate (P_N_) and transpiration rate (E) of five flag leaves were measured using a portable open-flow gas exchange system LI–6400 (Li–COR Inc., NE, USA) under 1200 μmol m^–2^ s^–1^ light intensity from a red/blue LED light source during 10:30–12:30 h on four consecutive days during the grain filling stage (16–19 August in 2011 and 19–22 August in 2012). The canopy temperature and humidity were monitored with HOBO dataloggers (HOBO U23-001 Pro Temp/RH, Onset Computer Corporation, Bourne, MA, USA) during anthesis. The dataloggers were installed in the top layer of the canopy in the center of the plot, and were located in the middle position of panicles [16]. Data were automatically recorded every 30 minutes from 8:00 to 20:00 h. The diurnal changes in canopy temperature and humidity were averaged as daily mean data. 

Economic benefit (US$ ha^–1^) was also roughly assessed; the output was grain yield (kg ha^–1^), and the investments mainly consisted of water consumption (m^3^ ha^–1^), seed rate (kg ha^–1^), consumption of plastic film (kg ha^–1^), and equipment consumption of drip irrigation system (US$ ha^–1^). Economic benefit (US$ ha^–1^) = grain yield (kg ha^–1^) × 0.52 US$ kg^–1^ – water consumption (m^3^ ha^–1^) × 0.07 US$ m^3^ – seed rate (kg ha^–1^) × 1.31 US$ kg^–1^ – 120 US$ ha^–1^ (consumption of plastic film) – 250 US$ ha^–1^ (equipment consumption of drip irrigation system) 

### Statistical analysis

All data were analyzed using the generalized linear model (GLM) procedure (SPSS16.0). Differences between means were compared by Fisher’s least-signiﬁcant-difference (LSD) test at the 5% probability level. 

## Results

### Weather and hydrological conditions

**Figure 2:**
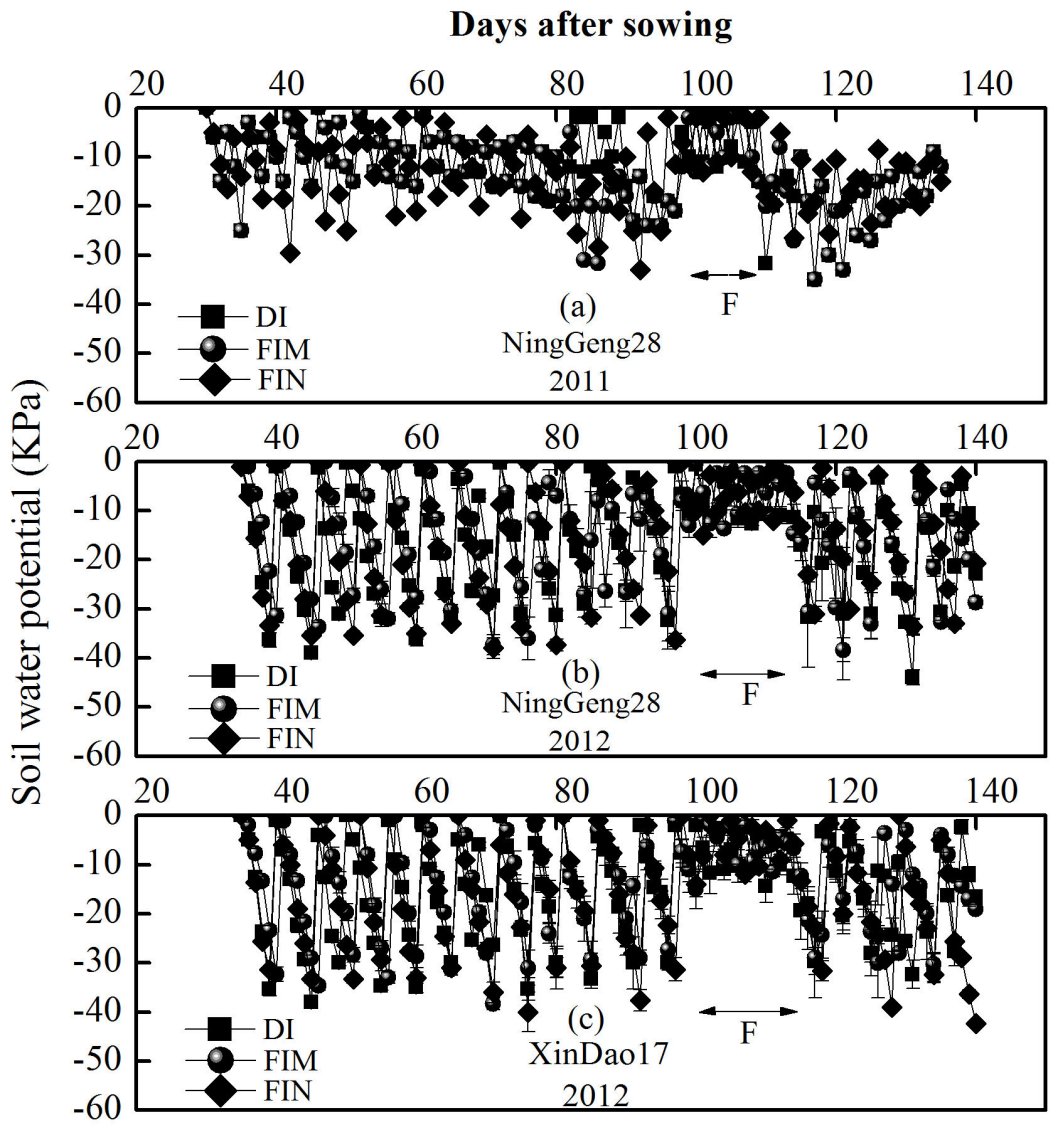
Hydrological dynamics of two rice cultivars under the non-flooded irrigation treatments (the DI, FIM, and FIN treatments). Cultivar Ninggeng28 (japonica) (a, b) and cultivar Xindao17 (japonica) (c) were grown in fields in Shihezi in 2011 and 2012. Vertical bars represent ±S.E. of the mean (n=3). F represents the flowering stage. Abbreviations are same as Figure 1.

During the rice growing period, the mean temperature, amount of solar radiation, and total rainfall were 22.42 °C, 3686.01 MJ m^-2^, and 105.53 mm, respectively, in 2011; and 21.14 °C, 3594.35 MJ m^–2^, and 63.91 mm, respectively, in 2012 (Table 1).The soil water dynamics in the non-flooded irrigation are shown in Figure 2. The soil water potential at a depth of 20 cm in the non-flooded irrigation treatments usually fluctuated between 0 and –30 KPa in 2011 and 2012, and it occasionally dropped to –40 KPa. The soil water potential ranged from 0 KPa to –10 KPa during flowering stage.

**Table 1:** Monthly means for daily air temperature, daily solar radiation and total rainfall during the rice growing period in Shihezi in 2011 and 2012.

	Mean temp. (°C)			Solar rad.(MJ m^–2^ d^–1^)			Rainfall (mm)	
	2011	2012		2011	2012		2011	2012
May	19.71	20.72		22.90	23.15		34.41	10.52
June	26.45	26.14		24.83	24.38		8.12	10.40
July	28.22	26.32		24.19	23.19		4.83	21.62
August	26.48	24.51		20.70	19.96		38.91	7.43
September	21.04	19.87		16.71	16.02		0	8.02
October	12.56	9.28		12.02	11.88		19.29	14.22

### Dry matter, LAI, tiller dynamic characteristics, and root morphology

**Figure 3:**
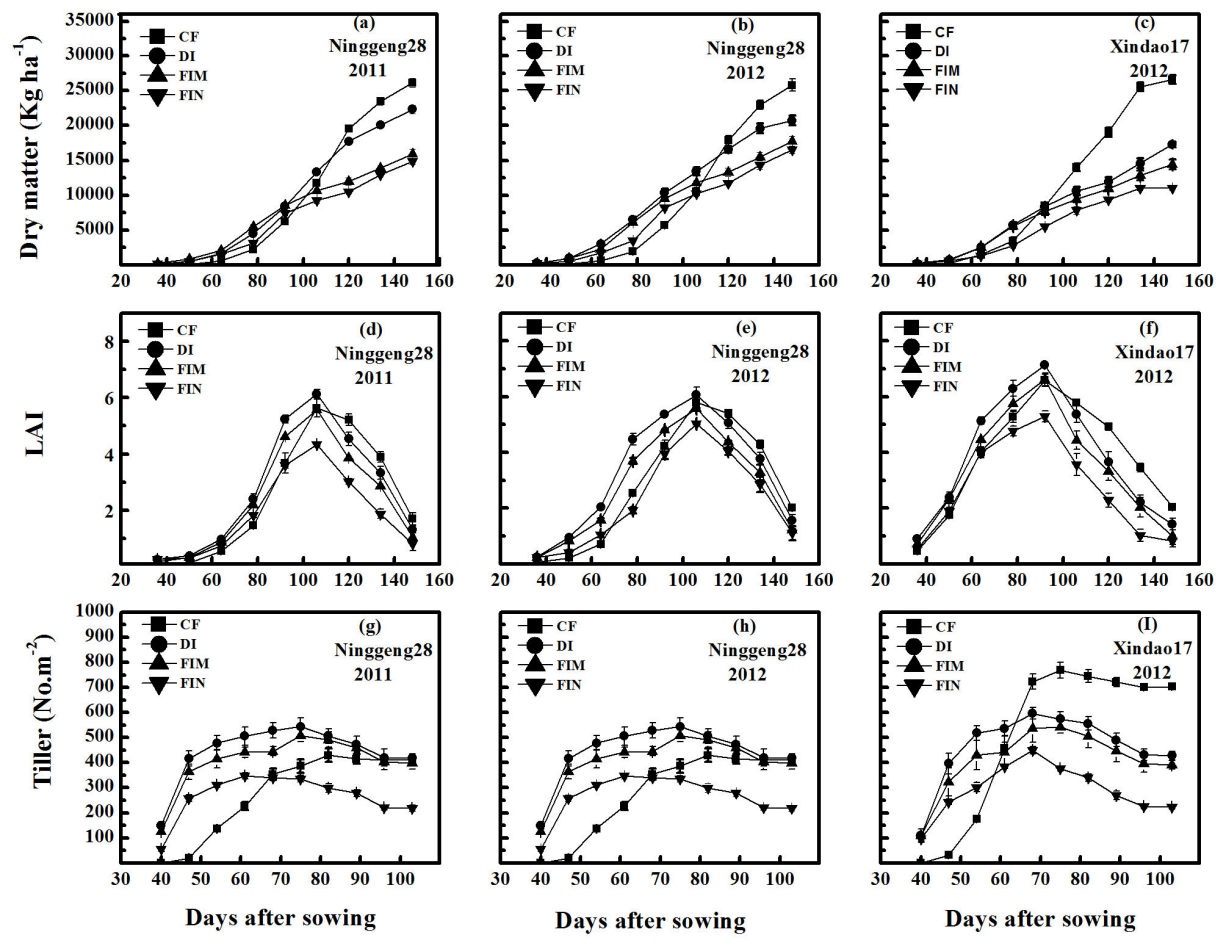
Dry matter, leaf area index (LAI), and tiller of two rice cultivars under the all irrigation treatments (the CF, DI, FIM, and FIN treatments). Dry matter (a, b, and c), LAI (d, e, and f), and tiller (g, h, and i) of cultivar Ninggeng28 (japonica) (a, b, d, e, g, and h) and cultivar Xindao17 (japonica) (c, f, and i) in 2011(a, d, and g) and 2012 (b, c, e, f, h, and i). Vertical bars represent ±S.E. of the mean. The S.E. of dry matter, LAI and tiller were calculated across twelve replicates, twelve replicates and thirty replicates in two cultivars, respectively. The CF indicates conventional flooding. Other abbreviations are same as Figure 1.

**Figure 4:**
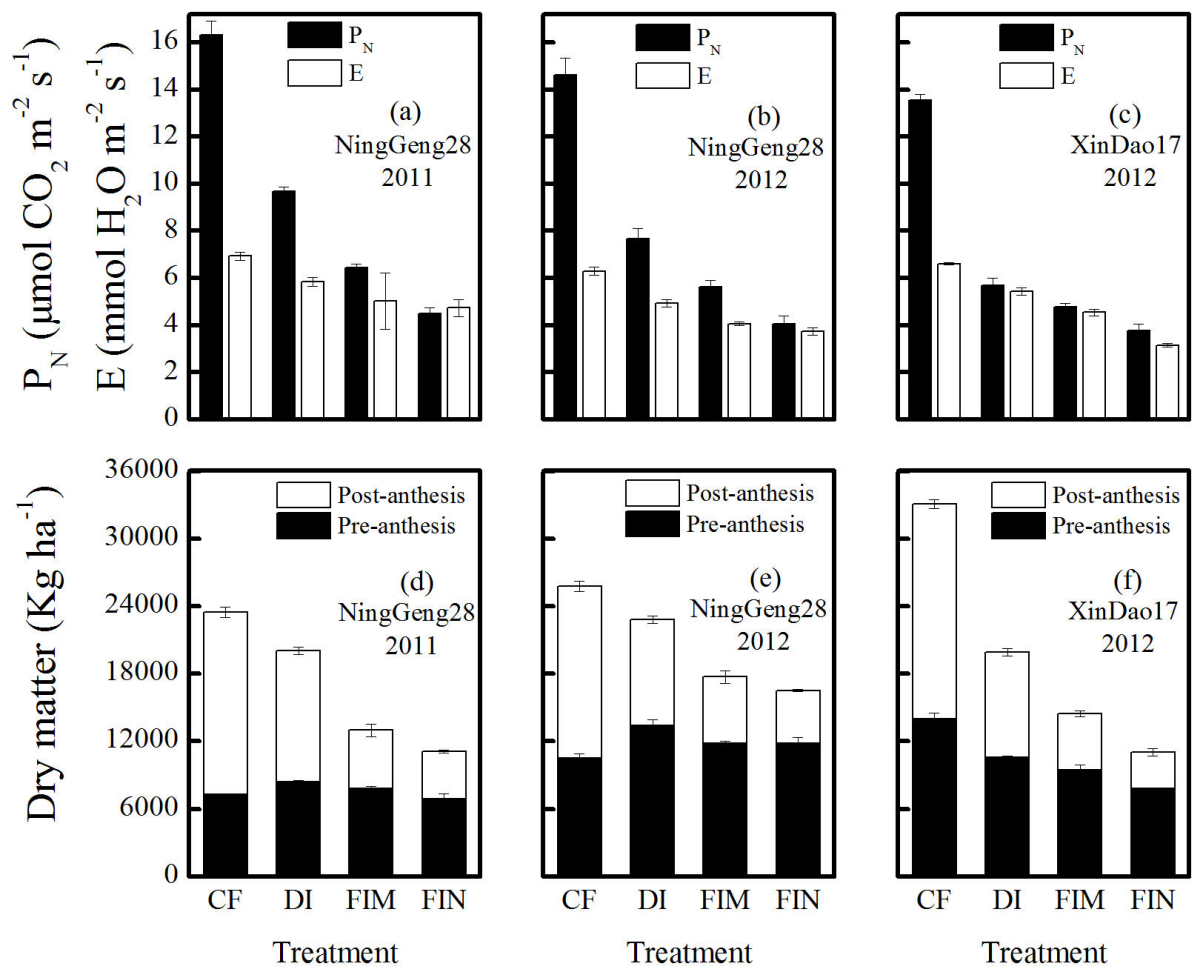
Photosynthetic rate at grain filling stage and dry matter of pre-anthesis and post-anthesis of two rice cultivars under the all irrigation treatments (the CF, DI, FIM, and FIN treatments). Photosynthetic rate (a, b, and c) and dry matter of pre-anthesis and post-anthesis (d, e, and f) of two rice cultivars of cultivar Ninggeng28 (japonica) (a, b, d, and e) and cultivar Xindao17 (japonica) (c, f) in 2011 (a, d) and in 2012 (b, c, e, and f). The S.E. of photosynthetic rate was calculated across five replicates for each day and averaged for the 4 days (from august16 to 19 in 2011 and august19 to 22 in 2012, respectively.), and the S.E. of dry matter of pre-anthesis was calculated across twelve replicates, the dry matter accumulation post-anthesis was calculated by dry matter at maturity – dry matter at anthesis from each plot (n=3). Abbreviations are same as Figure 1and Figure 3.

Dry matter production in the non-flooded irrigation treatments was greater than in the CF treatment during the vegetative phase, which was before 110 days after sowing for cultivar Ninggeng28 in both years and before 80 days after sowing for cultivar Xindao17 in 2012. But dry matter production was then consistently lower in the non-flooded irrigation treatments than in the CF treatment until maturity for the two cultivars (Figure 3a, b, and c). In short, dry matter accumulation in the non-flooded irrigation treatments markedly increased or only slightly decreased before anthesis, but significantly decreased after anthesis compared with the CF treatment across cultivars and years (Figure 4d, e, and f). Finally, the final dry matter in the CF treatment was 25.80–26.58 × 10^3^ kg ha^–1^ across cultivars and years, which was 19.63–64.96% more than that in the non-flooded irrigation treatments (Figure 3a, b, and c). Under non-flooded irrigation, dry matter production in the DI treatment was always higher than that in the FIM and FIN treatments for the two cultivars used during the rice-growing period in two years. A higher total dry matter production was recorded for cultivar Ninggeng28 than for cultivar Xindao17 under non-flooded irrigation in 2012 (Figure 3b, c). In addition, both the CF and DI treatments had significantly greater MT and MTE than the FIM and FIN treatments, no significant difference was observed between the CF treatment and the DI treatment for the MT and MTE parameters. The CPATG was 23.49–29.52% in the DI treatment, 16.13–16.34% in the CF treatment, 12.36–13.31% in the FIM treatment, and 0 in the FIN treatment, considering across both cultivars in 2012 (Table 2). 

**Table 2:** Invalid tillers, spikelets per unit area (sink capacity), matter translocation (MT), matter translocation efficiency (MTE), and contribution of pre-anthesis assimilates to grain weight (CPATG) under different cultivation conditions in two cultivars in Shihezi in 2012.

	Invalid tillers	Spikelets	MT	MTE	CPATG
	(No. m^–2^)	(10^3^ m^–2^)	(10^3^ Kg ha^–1^)	(%)	(%)
Ninggeng28					
CF^†^	96.81	60.85	1.38	19.32	16.13
DI	149.18	42.34	1.36	17.04	23.49
FIM	189.21	24.87	0.45	6.21	12.36
FIN	252.13	19.49	–0.32	–4.87	0
LSD (%)	23.25	16.38	0.36	2.33	3.59
Xindao17					
CF	112.54	102.41	1.48	21.49	16.34
DI	190.11	18.55	1.28	19.01	29.52
FIM	215.26	19.92	0.47	7.85	13.31
FIN	240.62	14.09	–0.43	–8.34	0
LSD (5%)	48.46	33.12	0.25	3.02	4.01

^†^ CF, DI, FIM, and FIN indicate conventional flooding cultivation, plastic film mulching with drip irrigation, plastic film mulching with furrow irrigation, and no mulching with furrow irrigation, respectively.

In the non-flooded irrigation treatments, the LAI was slightly higher than that in the CF treatment before anthesis, except for the FIN treatment, considering across cultivars and years (Figure 3d, e, and f). The peak of LAI was reached at flowering (nearly 100 days after sowing) for all treatments (Figure 3d, e, and f). The maximum LAI was 6.06–7.13 for the DI treatment, 5.63–6.59 for the CF treatment, 5.57–6.62 for the FIM treatment, and only 4.34–5.28 for the FIN treatment across the two cultivars and years. After anthesis, the CF treatment maintained the greatest LAI among treatments, and then followed by the DI, FIM, and FIN treatments in turn (Figure 3d, e, and f). 

The dates at which tillering began and maximum numbers of tillers reached were earlier for the non-flooded irrigation treatments than for the CF treatment across the cultivars and years (Figure 3g, h, and i). The final tiller numbers for cultivar Ninggeng28 was 410.38–420.59 plants m^-2^ for the CF treatment and 242.63–418.01 plants m^–2^ for the non-flooded irrigation treatments in both years (Figure 3g and h). For cultivar Xindao17, the final tiller number was 552.72 plants m^–2^ for the CF treatment, which was 29.04% greater than the tiller number in the DI treatment (Figure 3i). If the final tiller number was calculated as the number of tillers per main stem production, the tiller number was much higher in the CF treatment than in the non-flooded irrigation treatments for both cultivars (2.05–2.76 and 0.82–1.51 tillers per main stem for the CF treatment and for the non-flooded irrigation treatments, respectively). This shows that the CF treatment had higher tillering than the non-flooded irrigation treatments. Although little difference was observed for the final tiller number between the CF treatment and the plastic mulch treatments (the DI and FIM treatments), the plastic mulch treatments had greater invalid tiller rate than the CF treatment, and the FIN treatment had the highest invalid tiller among treatments (Table 2). Under the non-flooded irrigation treatments, both tillering ability and tiller numbers in the plastic mulching treatments (DI and FIM) during the tillering phase were markedly higher than that in the FIN treatment for cultivar Ninggeng28 in both years and for cultivar Xindao17 in 2012, but no significant difference was observed between the DI and FIM treatments across cultivars and years (Figure 3g, h, and i). Thus, plastic mulching appears to promote tiller capacity.

**Figure 5:**
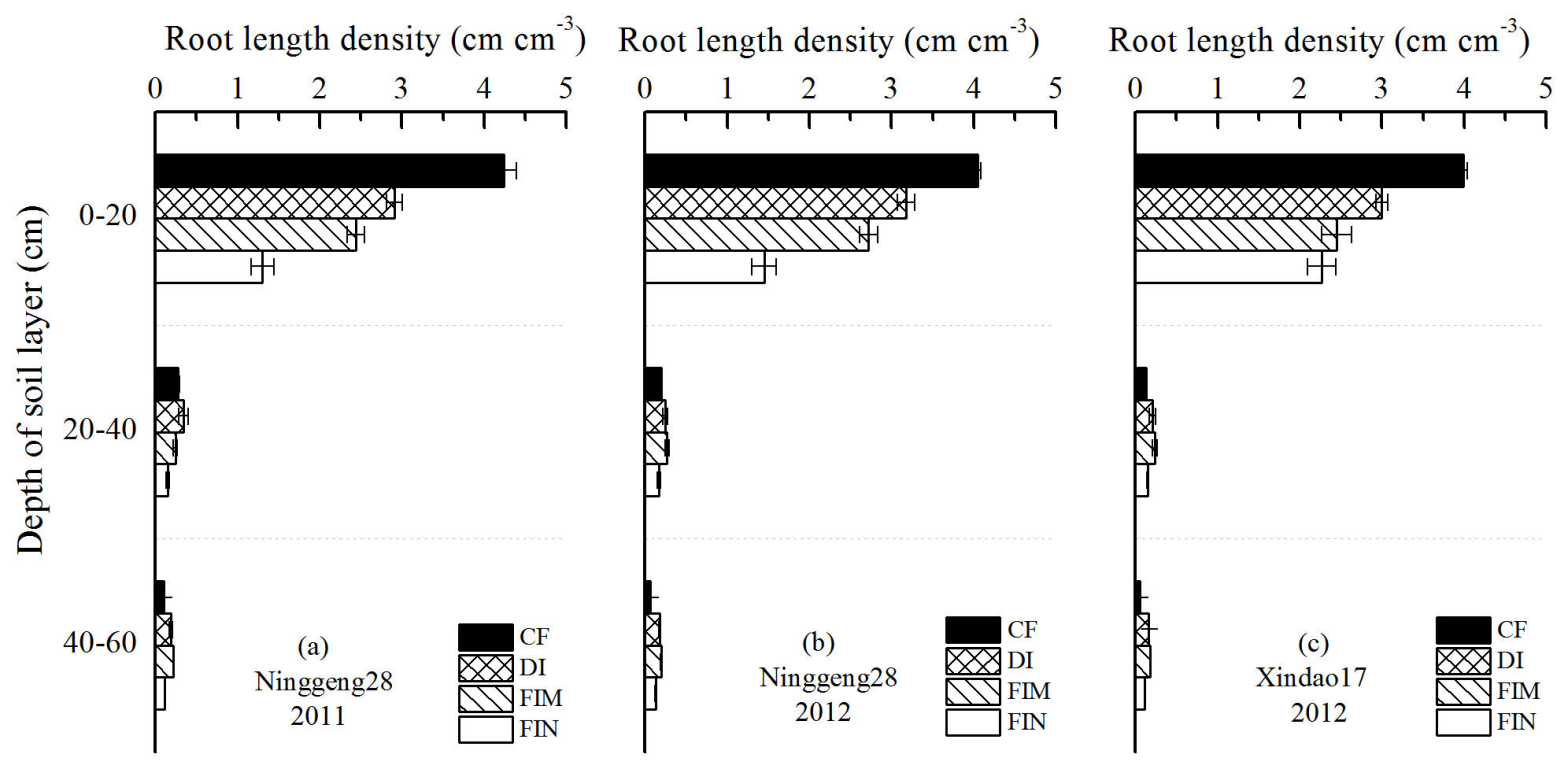
Root length density at flowering of two rice cultivars under the all irrigation treatments (the CF, DI, FIM, and FIN treatments). Root length density at flowering (1 August in 2011, and 4 August in 2012) of cultivar Ninggeng28 (japonica) (a, b) and cultivar Xindao17 (japonica) (c) in 2011 (a) and 2012 (b, c). Vertical bars represent ±S.E. of the mean (n=3). Abbreviations are same as Figure 1 and Figure 3.

Root length density (RLD) in terms of root length per unit soil volume decreased significantly with increasing depth of the soil layers across cultivars and years (Figure 5). For the non-flooded irrigation treatments, RLDs in the 0–20 cm soil layer were 1.45–3.18 cm cm^–3^, which were 21.48–62.08% less than in the CF treatment, but non-flooded irrigation treatments had a higher RLD than the CF treatment in the 20–40 cm and 40–60 cm soil layers across cultivars and years. Compared with the FIM and FIN treatments, the DI treatment significantly increased RLD in the 0–20 cm soil layer for both cultivars in both years.

### Yield and its components, water consumption, water use efficiency (WUE), and harvest index (HI)

In the DI treatment, the number of effective panicles per unit area was slightly higher than in the CF treatment, which both the DI and CF treatments were significantly higher than the FIM and FIN treatments. No significant differences were observed between treatments for cultivar Ninggeng28 in both years, but only accounted for 25.81% of the effective panicles compared with the CF treatment for cultivar Xindao17 in 2012 (Table 3). The spikelets per panicle, filled grain, and 1000-grain weight were 148.58–160.92, 77.03–86.54%, and 20.85–25.32 g, respectively, in the CF treatment, considering across cultivars and years, which the values of these parameters significantly decreased in turn among the DI, FIM, and FIN treatments compared with the CF treatment. Finally, grain yield was 8326.38–9040.27 kg ha^-1^ in the CF treatment, 3334.57–5901.61 kg ha^-1^ in the DI treatment, 2523.83–3576.42 kg ha^-1^ in the FIM treatment, and 2076.51–2313.52 kg ha^-1^ in the FIN treatment for both cultivars in two years. In generally, cultivar Ninggeng28 had higher yield and its components than cultivar Xindao17 (Table 3). 

**Table 3:** Grain yield and its components, water use efficiency (WUE, grain yield per amount of water supply, amount of irrigation plus amount of rainfall), harvest index (HI), and economic benefit under different cultivation conditions in two cultivars in Shihezi in 2011 and 2012.

		Effective	Spikelets	Filled	1000	Grain	Water	Water use	Harvest	Economic
		panicles	per	grain	grain	yield	consumption	efficiency	index	benefit
		(No.m^–2^)	panicle	(%)	weight (g)	(Kg ha^–1^)	(m^3^ ha^–1^)	(Kg m^–3^,)		($ ha^–1^)
2011	NingGeng28									
	CF	370.81	160.92	86.54	25.32	8326.38	34204.80	0.25	0.47	1863.43
	DI	411.82	108.82	74.13	22.03	5785.25	11215.02	0.51	0.42	1788.04
	FIM	242.22	83.14	49.12	21.13	3576.42	13585.31	0.25	0.15	795.53
	FIN	262.31	88.11	32.86	20.21	2076.51	13530.55	0.16	0.12	39.41
	LSD (5%)	58.43	13.87	9.91	0.71	526.44	1114.67	0.07	0.07	
2012	NingGeng28									
	CF	374.55	158.21	88.87	25.13	8579.85	35525.03	0.25	0.47	1902.27
	DI	399.01	102.81	72.06	22.25	5901.61	11030.18	0.53	0.41	1843.49
	FIM	235.51	81.04	50.87	20.04	3697.42	12079.12	0.31	0.14	963.88
	FIN	272.38	94.61	30.16	19.11	2095.51	13725.13	0.15	0.12	45.67
	Mean	320.36	109.17	60.49	21.63	5068.62	18089.81	0.31	0.28	
	LSD (5%)	115.31	8.83	14.01	1.21	507.78	375.91	0.06	0.09	
	XinDao17									
	CF	539.23	148.58	77.03	20.85	9040.27	34021.35	0.27	0.52	2250.12
	DI	198.87	104.31	43.99	17.23	3334.57	11306.39	0.38	0.17	831.91
	FIM	177.19	100.53	32.27	16.91	2523.83	12809.11	0.28	0.15	305.13
	FIN	172.78	81.54	26.56	15.64	2313.52	14244.11	0.16	0.13	95.31
	Mean	355.14	117.81	44.96	17.66	4803.04	18095.24	0.27	0.24	
	LSD (5%)	49.75	7.85	6.49	0.64	733.42	608.27	0.08	0.13	
	Variety	ns**^*a*^**	ns	*** **^*b*^**	***	*	ns	*	ns	
	Water regime	***	***	***	***	***	***	***	***	
	Variety×Water	***	*	*	***	***	ns	***	***	

CF, DI, FIM, and FIN indicate conventional flooding cultivation, plastic film mulching with drip irrigation, plastic film mulching with furrow irrigation, and no mulching with furrow irrigation, respectively. Grain weight is express at 14% moisture content.

^*^ represent significance at the 0.05 probability level.

^**^ represent significance at the0.01 probability level.

^***^ represent significance at the 0.001 probability level.

ns, nonsignificant at the 0.05 probability level.

 Irrigation water consumption ranged from 11030.18 to 14244.10 m^3^ ha^–1^ under the non-flooded irrigation treatments, but water consumption ranging from 34021.35 to 35525 m^3^ ha^–1^ was recorded in the CF treatment across cultivars and years. WUE in the DI treatment varied from 0.38 to 0.53 kg grain m^–3^ water, which was 1.41–2.12 times higher than that in the CF treatment, 1.35–1.89 times higher than that in the FIM treatment, and 2.37–3.78 times higher than that in the FIN treatment (Table 3). In addition, cultivar Ninggeng28 had a higher WUE than cultivar Xindao17, indicating that the DI system can greatly improve WUE, especially for cultivar Ninggeng28. The HI for the CF treatment was 0.47 for cultivar Ninggeng28 in both years, and slightly higher than that for the DI treatment, although the difference was not significant. However, the HI of cultivar Xindao17 in the DI treatment was significantly less than that in the CF treatment (0.52 for the CF treatment and 0.15 for the DI treatment) (Table 3). Under non-flooded irrigation treatments, the DI treatment had a significantly higher HI than the FIM and FIN treatments for both cultivars (Table 3).

### Main stem and tiller yield components, contribution to yield, and harvest index (HI)

Table 4 shows that the main stem contribution to yield was 31.43–50.18% for the CF treatment and 78.63–90.09% for the non-flooded irrigation treatments across both cultivars and two years, indicating that yield formation mainly depended on tiller panicles in the CF treatment and on main stem panicles for the non-flooded irrigation treatments. Yield, yield components, and HI of both main stem and tiller panicles were significantly higher for the CF treatment than for the non-flooding irrigation treatments, but the differences in main stem yield components and HI among the water treatments were less than differences in tiller panicles for the two cultivars used in both years. Thus, a low grain yield under the non-flooded irrigation treatments is dominantly attributed to poor performance of the tiller panicles. Because of the proportion of effective panicles, spikelets per panicle, and filled grain of tiller panicles sharply declining in the DI treatment for both cultivars in both years compared with the CF treatment, the grain yield and HI were then significantly reduced (Table 4). Under non-flooded irrigation, the DI treatment had the highest HI, grain yield and its components, and the FIN had the lowest HI, grain yield and its components for both main stem and tiller panicles across cultivars and years (Table 4). These results show the DI system can apparently improve the harvest index, grain yield and its components for both main stem and tiller panicles under non-flooded irrigation.

**Table 4:** Yield components, yield contribution capacity, harvest index of main stem panicles and tiller panicles under different cultivation conditions in two cultivars in Shihezi in 2011 and 2012.

		Effective panicles	Spikelets	Filled grain	1000-grain	Grain yield	Yield contribution	Harvest
		(No.m^–2^)	per panicle	(%)	weight (g)	(g hill^–1^)	capacity (%)	index
2011	Main stem							
	CF^†^	131.17	213.79	87.81	25.79	20.03	50.18	0.59
	DI	282.05	121.41	79.77	22.43	14.31	78.63	0.41
	FIM	237.15	106.75	65.01	20.91	5.73	81.44	0.22
	FIN	212.55	82.11	31.25	20.88	5.71	87.69	0.22
	LSD (5%)	23.91	7.53	10.39	0.76	2.57	14.76	0.03
	Tiller stem							
	CF	253.78	131.71	87.27	24.85	20.69	50.22	0.45
	DI	130.08	67.13	65.94	21.63	3.79	20.57	0.25
	FIM	35.14	32.11	34.96	18.47	0.85	6.33	0.03
	FIN	0	0	~	~	0	0	0
	LSD (5%)	52.71	22.36	10.57	0.68	4.29	4.76	0.06
2012	Main stem							
	Ninggeng28							
	CF	130.01	211.01	88.63	25.25	18.91	48.51	0.59
	DI	290.99	118.07	82.17	22.79	14.55	80.13	0.41
	FIM	240.55	103.53	64.68	20.97	5.72	83.84	0.22
	FIN	213.91	81.08	31.77	21.06	5.72	90.09	0.21
	LSD (5%)	62.18	25.47	18.25	1.58	3.43	28.89	0.08
	Xindao17							
	CF	148.89	214.94	78.57	20.63	18.11	31.43	0.67
	DI	172.78	108.29	56.34	17.78	4.92	87.21	0.26
	FIM	182.95	100.53	40.12	17.23	4.88	87.85	0.22
	FIN	157.54	80.43	27.12	15.64	4.15	87.81	0.21
	LSD (5%)	ns	12.73	20.16	0.77	2.92	27.58	0.11
	Tiller stem							
	Ninggeng28							
	CF	252.44	131.42	88.57	25.13	20.83	51.08	0.47
	DI	134.18	66.53	66.60	21.75	3.65	20.67	0.25
	FIM	35.96	31.32	35.85	18.53	0.91	6.17	0.03
	FIN	0	0	~	~	0	0	0
	LSD (5%)	58.22	28.32	24.54	3.04	3.61	11.31	0.08
	Xindao17							
	CF	493.44	135.10	75.48	21.08	30.13	62.47	0.51
	DI	1.33	47.24	19.29	15.12	0.18	1.69	0.01
	FIM	1.35	6.61	~	~	0.12	1.23	0.01
	FIB	1.01	4.01	~	~	0.09	1.07	0.01
	LSD (5%)	22.53	17.97	2.94	0.41	3.97	3.89	0.11

^†^ CF, DI, FIM, and FIN indicate conventional flooding cultivation, plastic film mulching with drip irrigation, plastic film mulching with furrow irrigation, and no mulching with furrow irrigation, respectively.

### Photosynthetic rate (P_N_), transpiration rate (E), and canopy temperature and relative humidity

**Figure 6:**
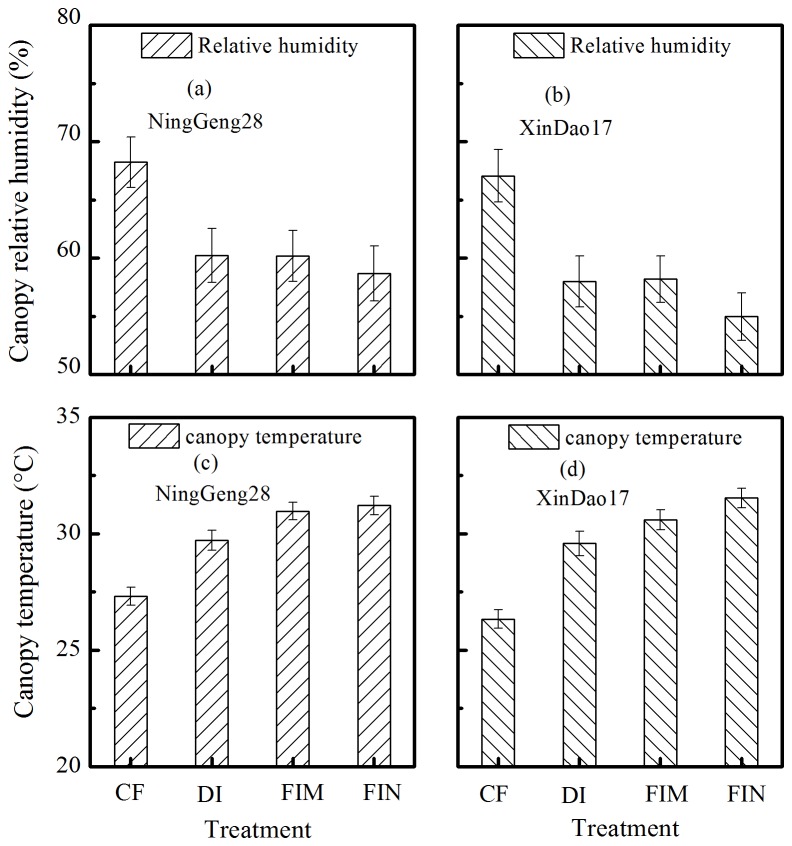
Canopy humidity and canopy temperature at flowering of two rice cultivars under the all irrigation treatments (the CF, DI, FIM, and FIN treatments). Canopy humidity (a, b) and canopy temperature (c, d) at flowering (from 28 July to 11 August) of cultivar Ninggeng28 (japonica) (a, c) and cultivar Xindao17 (japonica) (b, d) in 2012. Vertical bars represent ±S.E. of the mean, n=15 for canopy temperature and canopy humidity. Abbreviations are same as Figure 1and Figure 3.

The P_N_ of the CF treatment was 13.53–16.32 μmol m^–2^ s^–1^ across cultivars and years, which was 34.37–58.09% higher than that of the DI treatment, 60.48–61.56% higher than that of the FIM treatment, and 72.55–73.03% higher than that of the FIN treatment (Figure 5a, b, and c). The E was similar to that observed for the P_N_ across cultivation mode (Figure 5a, b, and c). At around flowering stage in 2012, the canopy temperature was 26.52–27.31, 29.58–29.72, 30.59–30.97, and 31.21–32.13°C for the CF, DI, FIM, and FIN treatments, respectively, in the two cultivars used (Figure 6c, d). And the relative humidity ranged from 57.23% to 68.64% across cultivars and water regimes (Figure 6a, b).

### Economic benefit

Economic benefit was assessed from five aspects which were grain yield, water consumption, seed rate, consumption of plastic film, and equipment consumption of drip irrigation system. Table 3 shows that the CF treatment had the highest economic benefit, and then followed by the DI, FIM, and FIN treatments in turn. For cultivar Ninggeng28, economic benefit of the CF treatment was 1893.43–1932.27 US$ ha^–1^, which was reduced by 68.78–75.39US$ ha^–1^ for the DI treatment, by 968.79–1097.91 US$ ha^–1^ for the FIM treatment, and by 1854.02–1886.91 US$ ha^–1^ for the FIN treatment compared with the CF treatment. For cultivar Xindao17, economic benefit per hectare was 2280.12 US$ for the CF treatment, 831.91 US$ for the DI treatment, 305.13 US$ for the FIM treatment, and 95.31 US$ for the FIN treatment.

## Discussion

Dry matter accumulation is a basal condition for grain yield formation. In the non-flooded irrigation treatments, the aboveground total biomass was lower than in the CF treatment (Figure 3a, b, and c); in agreement with previous studies in aerobic rice system and GCRPSs [12,22,30]. Lower total dry matter under the non-flooded irrigation treatments mainly related to a reduction in the amount of dry matter accumulation after anthesis (Figure 4d, e, and f), which the reasons can be explained by a lower photosynthetic rate [20,31], (Figure 4a, b, and c) and LAI [32], (Figure 3d, e, and f) during grain filling in the non-flooded irrigation treatments than in the CF treatment. It is probably an important reason for the decline in grain yield in the non-flooded irrigation treatments, and is in agreement with previous studies showing that crop failure mainly results from low biomass accumulation during post-anthesis [20,33,34]. Under non-flooded irrigation, the DI treatment showed a significantly greater dry matter accumulation after anthesis than the FIM and FIN treatments in both cultivars (Figure 4d, e, and f), so the DI treatment has a better production potential for rice growing under non-flooded irrigation. 

Although grain yield formation mainly depends on biomass accumulation after anthesis, the aboveground biomass before anthesis also plays an important role in yield production [20,31], and may contribute 20–40% to the final crop yield [35]. In this study, aboveground biomass in non-flooded irrigation was a little difference with the CF treatment (Figure 4d, e, and f), indicating non-flooded irrigation cultivation could not apparently affect dry matter accumulation before anthesis, especially for the DI treatment. However, both MT and MTE were significantly lower in the FIM and FIN treatments than in the CF and DI treatments, and no significant difference existed between the CF treatment and the DI treatment for these parameters (Table 2). Finally, the contribution of dry matter before anthesis (CPATG) to grain yield was only 0–13.31% in the FIM and FIN treatments across years and cultivars, which was significantly lower than that of the DI and CF treatments (Table 2). Thus, we deduce that the grain yield decrease could depend on the dry matter accumulation after anthesis in the DI treatment (Figure 4d, e, and f), and also be related to a low MT and MTE in the FIM and FIN treatments (Table 2). 

In the non-flooded irrigation treatments, grain weight was significantly lower than in the CF treatment (Table 3), integrating its lower filled grain trait (Table 3). This means that the grade of the filled grain was significantly reduced under the non-flooded irrigation treatments [36], which are caused by a reduction in the source supply capacity and/or by unimpeded characteristics of flow [37–39]. Our results showed that the DI treatment could actively transfer pre-anthesis accumulated dry matter (MT and MTE) to kernels to remedy a source decrease (Table 2), but the transformation capacity was very limited for the FIM treatment and even showed as a negative transformation for the FIN treatment when the source was in short supply in the non-flooded irrigation treatments during grain filling stage (Table 2). These results indicate that decrease in both the grain weight and filled grain could mainly be attributed to a source deficiency in the DI treatment, and that both the source and the flow could restrict grain weight and filled grain for the FIM and FIN treatments. Sink size (spikelets per unit area) showed a significant reduction compared with the CF treatment (Table 2), but it could be not a dominant factor limiting grain yield formation in this study because the source would still restrict the grade of the filled grain even if the sink size was expanded. Therefore, to improve grain yield under non-flooded irrigation, especially for the DI treatment, we should firstly increase the source supply capacity and then maybe amplify the sink size.

Also, the filled rate is directly affected by the ecological environment factors at anthesis, such as soil water content [29,40], the air temperature and relative humidity [41]. Which the soil water potential should be less than –10 KPa [29], the critical air temperature is 30 °C and a suitable relative humidity is 60–75% for most cultivars [41–43]. As shown in Figure 6, the temperature in this study ranged from 26.52 °C to 32.13 °C (Figure 6c and d) and the relative humidity was 57.22–68.64% (Figure 6a and b) across cultivars and water regimes in 2012. The air temperatures of the FIM and FIN treatments exceeded to 30 °C and were 3.12–5.61 °C higher than that of the DI and CF treatments, which the lower canopy temperature in the CF and DI treatments could be related to a higher transpiration rate characteristic compared with the FIM and FIN treatments (Figure 4a, b, and c). In addition, most of the treatments had relative humidity within a suitable range except for the FIN treatment. Thus, we infer that the ecological environment factors at anthesis could not be mainly reason decreasing percentage of ﬁlled grain in the DI treatment, while the high temperature around flowering could greatly affect the filled grain trait for the FIM and FIN treatments. These results also indicate that the DI treatment could maintain a favorable micro-ecological environment to meet the needs of rice growth under non-flooded irrigation.

RLD is an important indicator of potential water uptake [44], with a greater RLD implying a higher water extraction capacity [45]. Under non-flooded irrigation, the RLD in the 0–20 cm soil layer was significantly lower than under the CF treatment, but a greater RLD was observed in the 20–40 cm and 40–60 cm soil layers, except for the FIN treatment, across water regimes, cultivars, and years (Figure 5). These results are similar with previous studies in aerobic rice system and GCRPSs [16,21]. Large deep soil root systems play an important role in enhancing adaptability by absorbing nutrients and water from deep soil layers and thus easing water stress when top-soil roots are restricted by water stress [46]. Under non-flooded irrigation, the DI treatment had more roots in the topsoil than the FIM and FIN treatments for both cultivars (90-92.11% of total roots in the DI treatment and 86.53–88.42% of total roots in the FIM and FIN treatments, data not present), indicating that the root distribution zone in the DI treatment moved upwards under drip irrigation when compared with the furrow irrigation.

Tillering is an important population characteristic for grain yield formation in rice production [47]. Jiang found that the yield contribution from tillers ranged from 7% to 47% and mainly depended on the cultivars and water regimes [28]. In this study, the contribution of tillers to yield ranged from 0–20.67% with a greatly lower yield contribution from tillers under the non-flooded irrigation treatments than under the CF treatment across cultivars and years. This is attributed primarily to a reduction in the effective panicle number of tillers and a poor performance of the effective panicle number of tillers (Table 4). Under non-flooded irrigation, the invalid tiller was 33.24–60.68% more than that of the CF treatment for the two cultivars (Table 2). The considerable number of invalid tillers probably restricted high yield formation, which is supported by research evidence that the accumulated assimilate in invalid tillers is rarely transferred from invalid tillers to effective panicles and that much redundant matter and energy are greatly wasted when tiller panicles perform poorly. Finally, grain yield formation could be limited by inadequate material supply [48]. Alternatively, where there are large numbers of invalid tillers, the distribution of photosynthetically active radiation to effective panicles in the lower of canopy would be deteriorated [34], resulting in insufficient energy supply from the bottom leaves to the roots, and in turn restrict photosynthesis and assimilate accumulation [52]. Grain yield also could be affected because root functions were restricted at last [34]. Therefore, a reduction in invalid tillers and an increase in the productive tiller rate will apparently promote grain yield production under non-flooded irrigation [27]. This study also showed that improving productive tiller performance can clearly promote the agronomic traits of main stem panicles in non-flooded irrigation (Table 4), however, the reasons in regulating main stem and tiller panicles’ development are not clear, and it need to do follow-up study to find out scientific mechanisms.

The ground cover rice production systems under non-flooding irrigation including plastic film and straw mulching cultivation has been considered as a new water-saving technique and has high grain yield and WUE [13,15]. In this study, the plastic mulching treatments (DI and FIM) had a higher WUE than the FIN and CF treatments, but the CF treatment had the highest grain yield compared with the other treatments (Table 3). These results were supported by previous studies in aerobic rice and GCRPSs [12,16,49]. The yield gaps were 32–74.22% between the non-flooded irrigation and CF treatments, compared with a decline of only 0.70–21% in previous studies in which the threshold value of supplementary irrigation ranged from –25 KPa to –30 KPa during whole growing period [9,12,13,15,16]. The greater yield differences between the non-flooded irrigation and CF treatments in our study may be partly ascribable to ecotype differences, with previous trials conducted in semi-humid or humid regions [9,12,13,15,16,49]. However, our experimental field was located in arid region (Table 1). Yield decline amplitudes in dry environments are typically greater than in wet environments [9,50]. 

In this study, the plant density in the non-flooded irrigation treatments was 1.37 times higher than that in the CF treatment across cultivars and years (274.26 plants m^–2^ for the non-flooded irrigation treatments versus 200 plants m^–2^ for the CF treatment). The bad performance of the non-flooded irrigation treatments in grain yield and its components could be relevant to a higher plant density which had significantly affected source-sink relationship and population structure compared with the CF treatment (Figure 3, Table 4). However, when the threshold value of supplementary irrigation was –30 KPa before panicle initiation and –10 KPa from panicle initiation to maturity, and cultivation mode, plant density, and field management were the same as non-flooded irrigation in this study, the grain yield only decreased by 9.91% compared with the CF treatment (water regimes study in the DI treatment in 2012 for cultivar Ninggeng28, data not present). In addition, field investigation showed that no significant differences were observed for grain yield between the DI treatment and the CF treatment in 2011 and in 2012 (cultivation mode and plant density of the DI treatment were the same as non-flooded irrigation in this study, but more water and nitrogen were applied than in the DI treatment of this study. Personal communication with field management personnel). Therefore, we deduce the high plant density could not restrict grain yield and optimum cultivation mode, water, and nitrogen regimes would be key factors controlling high yield under the DI treatment. But the effects of high plant density on grain yield formation were not clear under the FIM and FIN systems and need to be verified under the current ecological conditions. Meanwhile, the future experiments also need to be carried out to find out the scientifically-sound theories for grain yield formation such as grain filling characteristics, photosynthetic productivity, source-sink relationship, etc. under non-flooded irrigation with high plant density. 

Under the non-flooded irrigation treatments, water consumption was reduced by 57.44–67.91% compared with the CF treatment (Table 3). The reductions in water consumption mainly resulted from decreasing seepage and evapotranspiration compared with the CF treatment [10,13,15,50]. The DI treatment had a higher water-saving capacity than the FIM and FIN treatments for the two cultivars in both years, with water consumption being the lowest for the DI treatment and being the highest for the FIN treatment among the non-flooded irrigation treatments (Table 3). The results could be supported by two reasons; on the one hand, plastic mulching can effectively reduce evapotranspiration compared with bare land [14,15,25,50] and on the other hand, the seepage was significantly lower in the DI treatment than in the furrow irrigation in a loamy soil at the same ecotype [51]. Because of the very low yield production in the FIN treatment, WUE was the lowest among treatments across the cultivars and years. Higher WUE was observed in the DI and FIM treatments, especially in the DI treatment, than in the CF treatment across cultivars and years (Table 3). Although the CF treatment had the highest economic benefit compared with the non-flooded irrigation treatments, only slight decrease in economic benefit occurred in cultivar Ninggeng28 in the DI treatment (Table 3), so it is not impossible to exceed the CF treatment by suitable varieties and reasonable water regimes (field investigation and another plot experiment in 2012 showed that the economic benefit of cultivar Ninggeng28 was greater under the DI system than under the CF system, data not present). These results suggest that the DI treatment can be considered a better water-saving cultivation technique in arid and semi-arid areas, particularly when accompanied by screening for suitable varieties and reasonable water regimes. 

## Conclusions

Although grain yield in the DI treatment was significantly lower than that in the CF treatment, WUE was 1.52-2.12 times greater in the DI treatment than in the CF treatment. Grain yield production mainly depended on tiller panicles in the CF treatment, but mainly on main stems in the non-flooded irrigation treatments, suggesting that the main reason was the poor performance in tiller panicles. More roots gathered in deep-soil layer in the non-flooded irrigation treatments than in the CF treatment. On the other hand, the DI treatment had a higher grain yield and HI, and more effective tillers, more roots in topsoil, higher WUE, and greater economic benefit compared with the FIM and FIN treatments. Therefore, the DI treatment could be considered a better water-saving cultivation technique in areas of arid and semiarid region.
